# Xbp1 Directs Global Repression of Budding Yeast Transcription during the Transition to Quiescence and Is Important for the Longevity and Reversibility of the Quiescent State

**DOI:** 10.1371/journal.pgen.1003854

**Published:** 2013-10-31

**Authors:** Shawna Miles, Lihong Li, Jerry Davison, Linda L. Breeden

**Affiliations:** 1 Basic Science Division, Fred Hutchinson Cancer Research Center, Seattle, Washington, United States of America; 2 Computational Biology, Public Health Science Division, Fred Hutchinson Cancer Research Center, Seattle, Washington, United States of America; University of Toronto, United States of America

## Abstract

Pure populations of quiescent yeast can be obtained from stationary phase cultures that have ceased proliferation after exhausting glucose and other carbon sources from their environment. They are uniformly arrested in the G1 phase of the cell cycle, and display very high thermo-tolerance and longevity. We find that G1 arrest is initiated before all the glucose has been scavenged from the media. Maintaining G1 arrest requires transcriptional repression of the G1 cyclin, *CLN3*, by Xbp1. Xbp1 is induced as glucose is depleted and it is among the most abundant transcripts in quiescent cells. Xbp1 binds and represses *CLN3* transcription and in the absence of Xbp1, or with extra copies of *CLN3*, cells undergo ectopic divisions and produce very small cells. The Rad53-mediated replication stress checkpoint reinforces the arrest and becomes essential when Cln3 is overproduced. The *XBP1* transcript also undergoes metabolic oscillations under glucose limitation and we identified many additional transcripts that oscillate out of phase with *XBP1* and have Xbp1 binding sites in their promoters. Further global analysis revealed that Xbp1 represses 15% of all yeast genes as they enter the quiescent state and over 500 of these transcripts contain Xbp1 binding sites in their promoters. Xbp1-repressed transcripts are highly enriched for genes involved in the regulation of cell growth, cell division and metabolism. Failure to repress some or all of these targets leads *xbp1* cells to enter a permanent arrest or senescence with a shortened lifespan.

## Author Summary

Complex organisms depend on populations of non-dividing quiescent cells for their controlled growth, development and tissue renewal. These quiescent cells are maintained in a resting state, and divide only when stimulated to do so. Unscheduled exit or failure to enter this quiescent state results in uncontrolled proliferation and cancer. Yeast cells also enter a stable, protected and reversible quiescent state. As with higher cells, they exit the cell cycle from G1, reduce growth, conserve and recycle cellular contents. These similarities, and the fact that the mechanisms that start and stop the cell cycle are fundamentally conserved lead us to think that understanding how yeast enter, maintain and reverse quiescence could give important leads into the same processes in complex organisms. We show that yeast cells maintain G1 arrest by expressing a transcription factor that represses conserved activators (cyclins) and hundreds of other genes that are important for cell division and cell growth. Failure to repress some or all of these targets leads to extra cell divisions, prevents reversible arrest and shortens life span. Many Xbp1 targets are conserved cell cycle regulators and may also be actively repressed in the quiescent cells of more complex organisms.

## Introduction

Budding yeast that are grown in rich glucose-containing media and are allowed to naturally exhaust their carbon source undergo a series of changes that enable a significant fraction of the cells, primarily daughter cells, to enter a protective quiescent (Q) state [1]. As yeast cells transition to quiescence, they shift to respiration [2] and stockpile their glucose in the form of glycogen and trehalose [3], [4]. These Q cells are significantly denser than their nonquiescent (nonQ) siblings, which enables us to purify them by density sedimentation [1]. The ability to purify Q cells offers a unique opportunity to study this transition.

An important characteristic of all quiescent cells is that they arrest their cell cycle in G1. This requires the G1 to S transition to be stably halted by a mechanism that can be readily reversed when conditions permit. In cycling cells, progression through G1 into the next S phase involves two consecutive waves of G1 cyclin (Cln) expression. *CLN3* is transcribed at the M/G1 border [5] and Cln3 associated with the cyclin-dependent kinase (Cdk) activates the transcription of the *CLN1* and *CLN2* cyclins and other genes that trigger budding and DNA replication [6]–[8]. If the fidelity or timing of S phase is disrupted, there are checkpoint proteins, including Rad53 and Rad9, which monitor incomplete or damaged DNA and delay cell division to allow for reparations [9].

Cln3/Cdk activity is rate limiting for the G1 to S transition during exponential growth. Excess Cln3 results in shorter G1 phases and smaller cells, while loss of Cln3 function prolongs G1 and results in larger cells [10], [11]. Previous studies have shown that the G1 cyclin Cln3, ectopically expressed during stationary phase from the *UBI4* promoter, prevents G1 arrest and causes loss of viability [12]. Tetraploid cells also die in stationary phase and this inviability can be completely rescued by deletion of all four *CLN3* genes [13]. These deleterious effects indicate that Cln3/Cdk must be tightly controlled during stationary phase and that its deregulation antagonizes entry into the Q state.

In this work, we demonstrate that G1 arrest is initiated before the diauxic shift (DS), which is when all the glucose has been scavenged from the media. *CLN3* is a critical target of repression for G1 arrest and for the transition to quiescence. Rad53 checkpoint activity reinforces this arrest in wild type cells and becomes essential when Cln3 is overproduced. Xbp1 is also important for maintaining G1 arrest. Xbp1 is a repressor of *CLN3* transcription [14], [15]. It is related to the Swi4/Mbp1 family of transcription factors, which are the DNA binding components of the yeast complexes paralogous to E2F/Dp1 in higher cells [7], [8]. As glucose is exhausted from the media, the *XBP1* transcript is induced and it is among the most abundant transcripts in Q cells. Xbp1 binds and represses hundreds of genes, including *CLN3* during the post-DS phase of growth. In the absence of Xbp1, cells undergo extra post-DS cell divisions and produce very small cells. These phenotypes are Cln3-dependent. *xbp1* mutant Q cells are also defective in the maintenance of and recovery from the Q state. *xbp1* Q cells maintain viability, but lose the ability to re-enter the cell cycle. Using Next Generation Sequencing [16], we have identified over 800 transcripts that are repressed three-fold or more by an Xbp1-dependent mechanism and 520 of these contain Xbp1 binding sites in their promoters. Xbp1 binds directly to all seven of the promoters we tested, in vivo, but only in post-DS cells. These findings indicate that Xbp1 is a global regulator specifically during the transition to quiescence. Xbp1's other targets include many genes involved in cell division, with a particular enrichment of genes required for cytokinesis. Many genes whose products localize to sites of polarized cell growth and are involved in cell wall remodeling are targeted by Xbp1. In addition, many metabolic and transport pathways are repressed by Xbp1.

## Results

### G1 arrest is initiated before the diauxic shift

**Figure 1:**
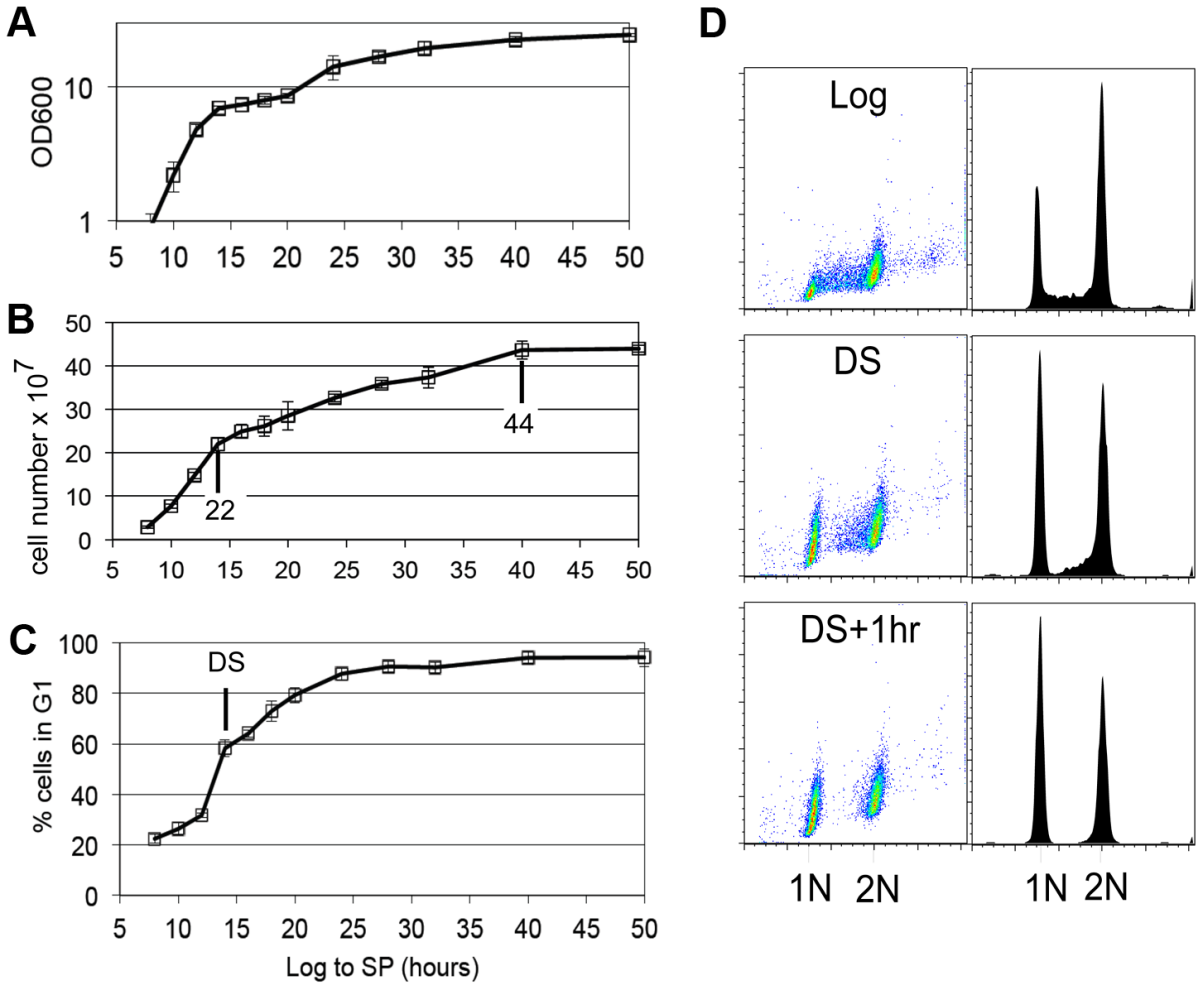
G1 arrest is initiated before the diauxic shift. (A) Culture density based on optical density at 600 nm wavelength (OD600), (B) cell number increase, (C) percentage of cells in G1 are plotted for wild type cells grown in YEPD medium from log to stationary phase (stationary phase.) Values are the average of four growth curves and error bars are included. Cell number after the DS (14 hr) and at the end of cell division are indicated. (D) DNA fluorescence shown as scatter plots and histograms of log phase wild type cells, cells at the DS, and one hour later.

Yeast cells spend most of their time in a non-dividing state triggered by nutrient depletion from their environment. Under the conditions we employ (see Methods), yeast undergo a highly reproducible transition from the logarithmic (log) phase of growth to stationary phase in response to carbon limitation. Figure 1 shows the average of four growth curves in which we monitored cell density, cell number and DNA content as prototrophic W303 cells grew from log phase to stationary phase in rich medium. The turbidity of the culture increases over this time course to an optical density (OD_600_) of about 24, but the cell number only doubles once after the DS, which occurs between the 12 and 14 hour time points. We have monitored the DNA content of these cells to determine what fraction of cells are in G1, S and G2/M over this time course. Interestingly, the 12 to 14 hour interval shows the sharpest increase in the percentage of cells in G1. This indicates that the signal to slow proliferation is occurring at or before the DS and cells respond by extending or arresting in G1. Figure 1D shows the DNA of wild type cells in log phase (8 hours), immediately after the DS, and one hour later. During log phase, the G1 (1N) and G2/M (2N) cells form two spots or peaks of high density by flow cytometry. The cells in S phase, with intermediate DNA content, are scattered between them and make up about 20% of the cells in the population. At the DS, the percentage of cells in G1 is already double that of log phase cells. This indicates that cells begin to slow the G1 to S transition before the diauxic shift. Also at the DS, we see a drop in the number of cells that are in early S phase (Figure 1D,) which is an indication that the initiation of new DNA synthesis ceases at this time. One hour after the DS, less than 3% of the cells are in S phase, and this pattern persists for at least 34 hours. We conclude that the signal to stop proliferation is received before the cells have scavenged all the glucose from the media and they respond by extending G1. The halt to DNA replication is correlated with and could be triggered by the DS.

### Stable G1 arrest involves down-regulation of Cln3 by Xbp1

**Figure 2:**
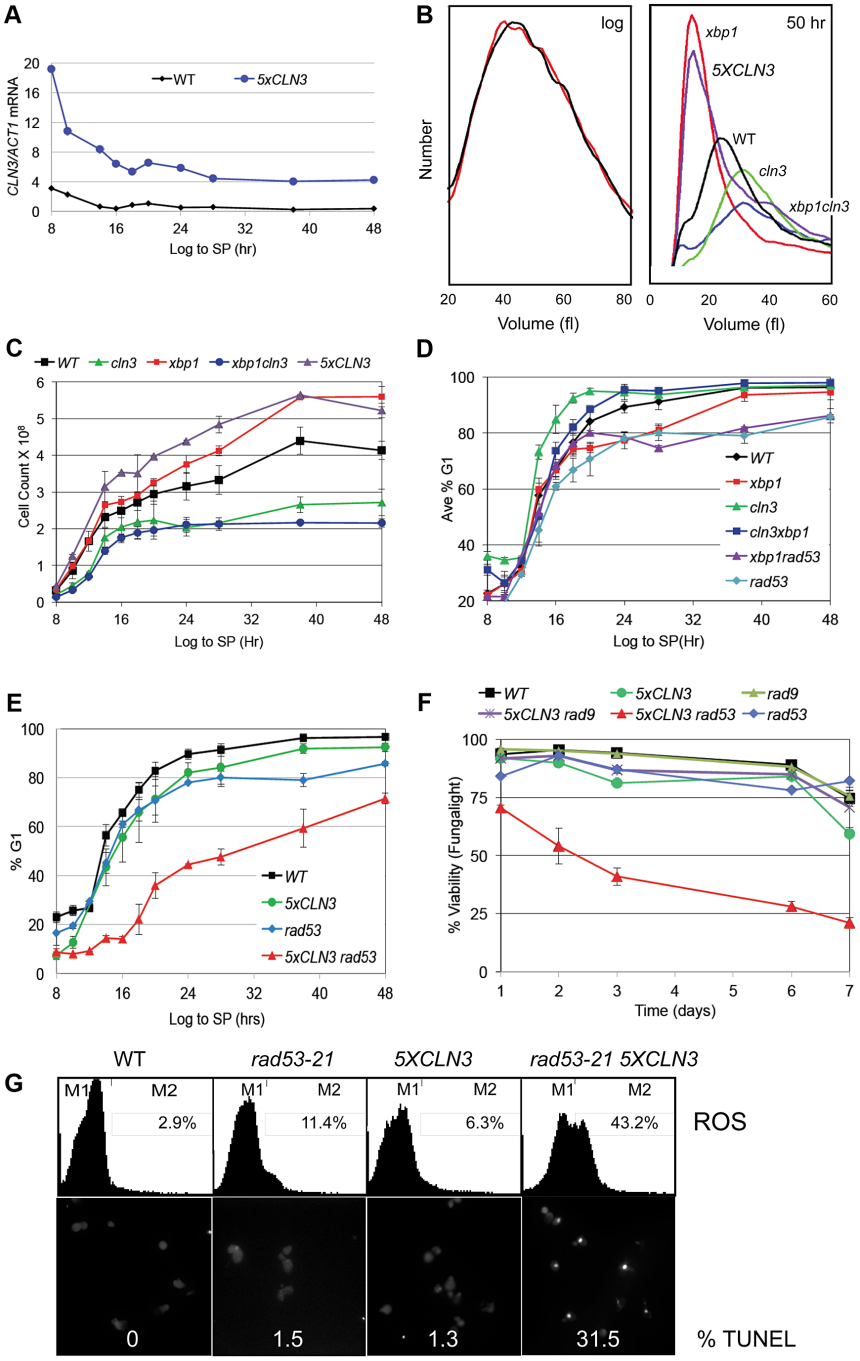
Xbp1 and Rad53 are required to stop cell division and maintain repression of *CLN3* transcription in stationary phase. (A) Transcript levels of *CLN3* and *ACT1* as cells grow for 8 to 48 hours into stationary phase (SP) were measured and reported as a ratio of *CLN3/ACT1*, (B) Cell volume distribution in log phase cultures (left) of *xbp1* (red) and wild type (black) cells and cultures that have ceased dividing after 50 hours of growth (right), relevant genotypes are indicated (C) Cell number, and (D and E) percent of cells in G1 as cells grow from log phase to SP. (F) Cell viability based on FungaLight dye exclusion in log phase cells (day 1) and after seven days of further growth into SP. (G) Upper panel, flow cytometry assays of ROS accumulation in strains indicated after five days (OD_600_ = 24) of growth into SP. Percent of ROS positive cells within the M2 gate are shown in upper right of each panel. Below are micrographs and quantification of TUNEL positive cells after five days of growth into SP. Relevant genotypes indicated (BY6500 WT, BY5654 *5XCLN3*, BY6602 *xbp1*, BY6873 *cln3*, BY7131 *cln3xbp1*, BY7146 *xbp1 rad53-21*, BY6848 *rad53-21*, BY6697 *rad53-21 5XCLN3, BY7406 rad9 5XCLN3*.).

To determine how G1 arrest is accomplished, we have assessed the role of several key regulators of the G1 to S transition. Cln3/Cdk activity is rate limiting for the G1 to S transition during exponential growth. To investigate the effects of over-producing Cln3 on Q cell formation, we generated a prototrophic strain carrying five copies of the wild type *CLN3* gene (*5XCLN3*). This strategy maintains all the regulatory features of the wild type *CLN3* gene, while it increases the Cln3 expression level. We first verified that the *5XCLN3* construct produces about five-fold higher levels of *CLN3* mRNA than wild type as cells grow from log to stationary phase (Figure 2A). To assess the impact of excess Cln3 on the transition to quiescence, we compared Q cell yield in *5XCLN3* cells to *cln3* mutant and wild type cells. *5XCLN3* consistently reduces Q cell yield by half, and *cln3* mutants increase Q cell production by at least 30%. This confirms that Cln3 activity is above wild type levels in the *5XCLN3* strain and that this excess Cln3 inhibits Q cell formation. It also suggests that cells enter the Q state from G1 and the longer they stay in G1 the more likely they are to achieve a successful transition into this state.

The *CLN3* transcript level is high in rapidly cycling cells then it drops abruptly as cells enter stationary phase (Figure 2A). This is not unexpected, because the *CLN3* promoter is cell cycle regulated [5], and it is activated by Azf1 in the presence of glucose [17], [18]. In addition, *CLN3* is a target of the Xbp1 repressor, which is highly induced by glucose limitation [14].

Xbp1 is a transcriptional repressor that is not expressed during the log phase of growth, but it is induced by many forms of stress, including DNA damage and glucose limitation [14], [19]. When Xbp1 is ectopically produced in log phase cells, it binds to and represses the *CLN3*, *CLN1* and *CLB2* cyclin promoters [15]. Xbp1 overproducers also grow slowly and prolong the G1 phase of the cell cycle [14], [20]. This led us to ask if Xbp1 could be important for repressing *CLN3* and halting cell division during the transition from log phase to quiescence. *xbp1* and wild type cells are identical in size during logarithmic growth, however *xbp1* cells are much smaller than wild type cells when grown to stationary phase (Figure 2B). This could be explained if *xbp1* mutants continue proliferating under growth limiting conditions and the physical growth of the resulting cells is impaired. Figure 2C shows that this is the case. *xbp1* cultures attain a higher cell number at stationary phase than do wild type cells, indicating that they undergo extra cell divisions. This can also be seen as a slower accumulation in G1 (Figure 2D). The *xbp1* mutant reaches 80% G1 eight hours after wild type cells. If *CLN3* is a critical target of Xbp1, we expected that the ectopic cell divisions, the small cell size, and the G1 arrest delay of *xbp1* mutants would depend on the presence of Cln3. We have assayed these phenotypes in the *xbp1cln3* double mutant. As predicted, *xbp1cln3* cells are the same large size as *cln3* cells (Figure 2B), and they undergo fewer cell divisions, as do *cln3* cells (Figure 2C.) *xbp1cln3* cells also display the same rate of accumulation in G1 that is seen in wild type cells (Figure 2D). This shows that these hyper-proliferative phenotypes of *xbp1* are Cln3-dependent. We also expected that *5XCLN3* would share these *xbp1* phenotypes. Figure 2B shows that *5XCLN3* cells are the same small size as *xbp1* cells during post-diauxic growth, they undergo extra cell divisions like *xbp1* (Figure 2C), and *5XCLN3* delays G1 arrest (Figure 2E.)

### Rad53 checkpoint activity reinforces arrest and promotes Q cell formation

During logarithmic growth, accelerating the transition from G1 to S causes a sub-optimal S phase and such cells cannot survive without eliciting the replication stress checkpoint [21]–[23]. The fact that excess Cln3 only delays G1 arrest led us to wonder if the replication stress checkpoint also plays a role in restraining cell cycle progression under these conditions. To test this, we combined *rad53-21*, which lacks checkpoint activity [24] with *5XCLN3*. These cells were grown from log phase into stationary phase and assayed for their ability to G1 arrest. Like *5XCLN3*, *rad53-21* alone has a modest G1 arrest defect. However, Rad53 is critically important for G1 arrest and Q cell formation when Cln3 is in excess. *rad53-21 5XCLN3* cells divide more slowly and undergo the DS six hours later than wild type cells. They very gradually accumulate in G1, reaching 50% G1 about 30 hours later than wild type (Figure 2E). These cells also lose viability rapidly as they enter stationary phase (Figure 2F.) After seven days of growth 80% of the *rad53-21 5XCLN3* cells were dead based on vital dye staining. *rad53-21 5XCLN3* cells are also completely defective in Q cell formation. These results indicate that the excess Cln3 produced by the *5XCLN3* loci is toxic to nutrient-limited cells that do not have Rad53 checkpoint function. It is worth noting that this experiment was carried out at a constant pH in rich medium. Therefore, this loss of viability cannot be due to acidification, as it is in unbuffered, minimal media [25].

Reactive oxygen species (ROS) and DNA fragmentation has been associated with DNA damage and replication stress in yeast and metazoan cells [26], [27]. During log phase, 8% of the *rad53-21 5XCLN3* cells were ROS positive (data not shown.) By day five, 43% of these cells contained ROS and 31% showed DNA fragmentation, as detected by TUNEL staining (Figure 2G). ROS was also detectable in *5XCLN3* and *rad53-21* single mutants, but they showed no detectable TUNEL positive cells and high viability over this time course, which indicates that they were able to tolerate this level of ROS accumulation. However, the *rad53-21* cells contained four times more ROS than wild type cells by day five (Figure 2G). This indicates that wild type cells also rely on Rad53 checkpoint activity during the transition to quiescence.

Rad53 is activated in response to both replicative stress and DNA damage. To see if DNA damage is involved, we combined *5XCLN3* with *rad9*, which is a DNA damage-specific checkpoint protein [28]–[30]. *5XCLN3* showed no toxicity in the absence of Rad9 (Figure 2F). We conclude that cells utilize the replicative stress checkpoint to reinforce cell cycle arrest during the transition to quiescence. Cells that fail to down-regulate *CLN3* during this transition depend on this checkpoint for their survival. Checkpoint failure leads to apoptotic cell death.

### Xbp1 binds the *CLN3* promoter *in vivo* and represses its transcription in post-diauxic cells

**Figure 3:**
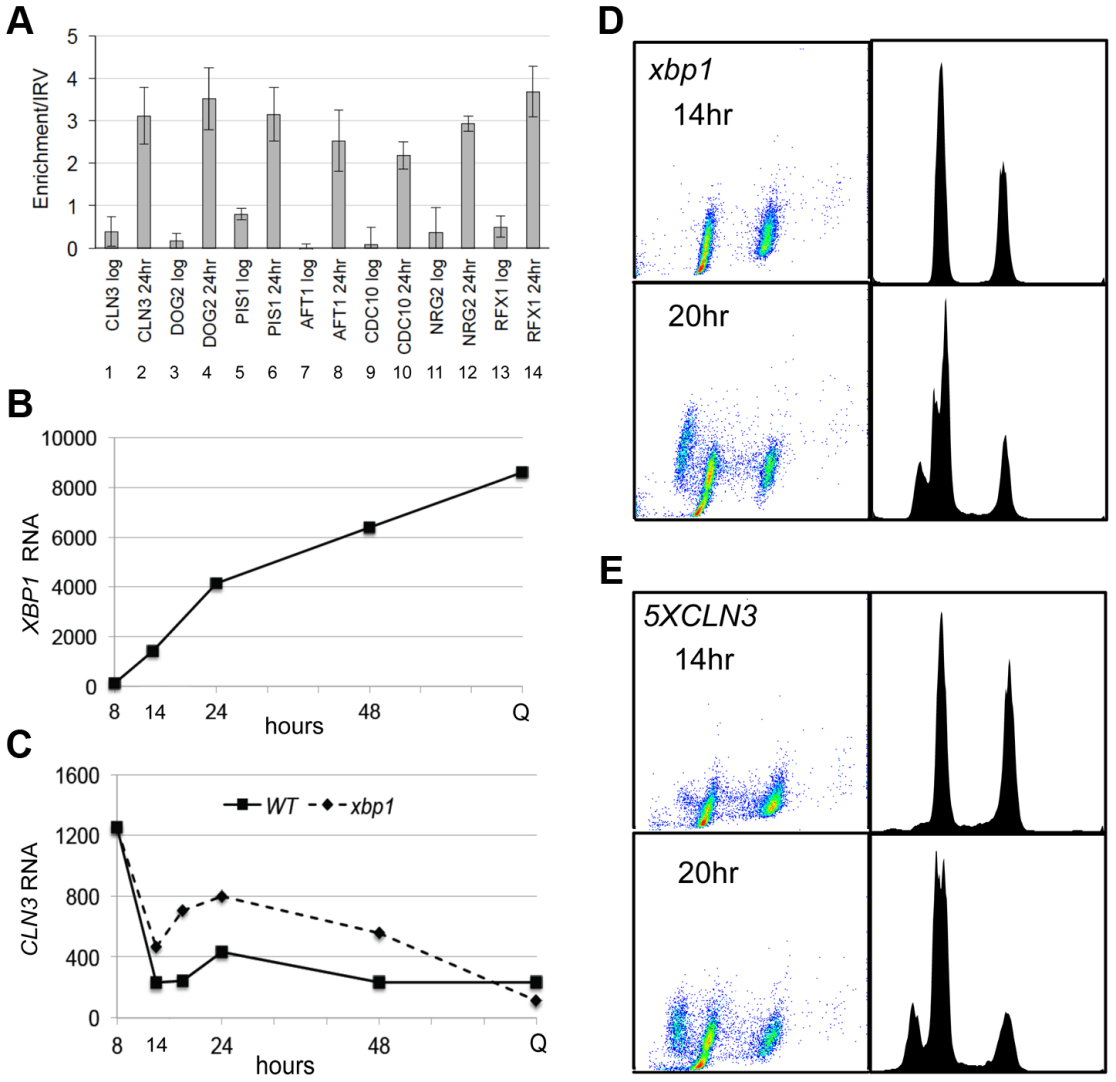
Xbp1 binds and represses *CLN3* transcription in post-diauxic cells. (A) ChIP was performed on the promoters indicated in log phase cells and after 24 hours of growth. (B) *XBP1* and (C) *CLN3* mRNA levels in wild type and *xbp1* cells grown from log phase to stationary phase quantified from Next-Generation RNA sequencing data. (D) *xbp1* and (E) *5XCLN3* cells harvested for FACS analysis as in Figure 1 after 14 and 20 hours of growth (BY6500 WT, BY5654 *5XCLN3*, BY6602 *xbp1*.).

Repression of *CLN3* is important for the G1 arrest that is initiated by glucose limitation, and our data are consistent with Xbp1 playing a role in that process. However, when we combine *xbp1* with *rad53-21*, there is no additive effect. The *xbp1 rad53-21* is no more defective in G1 arrest then *rad53-21* alone (Figure 2D). This suggests that Xbp1 may not repress *CLN3* under these conditions. To directly assess the role of Xbp1 in *CLN3* repression, we used chromatin immunoprecipitation and RNA Next Generation sequencing. Figure 3A (lanes 1 and 2) show that Xbp1 binding to the *CLN3* promoter is undetectable in log phase cells, but it is clearly bound in cells harvested after 24 hours of growth. This can be explained by the fact that Xbp1 is induced by glucose limitation. Figure 3B shows the dramatic induction of *XBP1* mRNA that begins before the diauxic shift (14 hours) and continues for 48 hours. It is also present at very high levels in Q cells purified from a seven day old culture. In fact, *XBP1* ranks within the top 100 most abundant transcripts in Q cells. Figure 3C shows *CLN3* mRNA levels over this same time course in wild type and *xbp1* cells. The initial pre-DS drop in *CLN3* mRNA still occurs, but we see a two to three-fold de-repression of *CLN3* from 14 to 48 hours in the absence of Xbp1. It then drops to a very low level in Q cells, and that drop is also Xbp1-independent. This pattern suggests that there may be three distinct mechanisms for establishing and maintaining *CLN3* repression and that Xbp1 plays a role in maintaining *CLN3* repression during post-diauxic growth.

The fact that the pre-DS drop in *CLN3* levels still occurs in *xbp1* cells indicates that the initial signaling to slow proliferation is intact. Figure 2C and D also show that cell number and the fraction of *xbp1* cells in G1 is very similar to wild type for the first 18 hours. Direct comparison of the FACS profiles of wild type (Figure 1D) and *xbp1* cells (Figure 3D) shows that *xbp1* cells halt S phase as well as wild type at the 14 hour time point, but by 20 hours a new S phase population has emerged (Figure 3D.) This S phase re-entry is also Cln3-dependent (data not shown.) In contrast, S phase cells are present at the DS and throughout this time course in the *5XCLN3* population (Figure 3E.) It is possible that either the timing or the extent of replication driven by *5XCLN3* makes these cells more dependent upon the Rad53 replication stress checkpoint for viability.

### Xbp1 is important for the longevity and reversibility of the Q state

**Figure 4:**
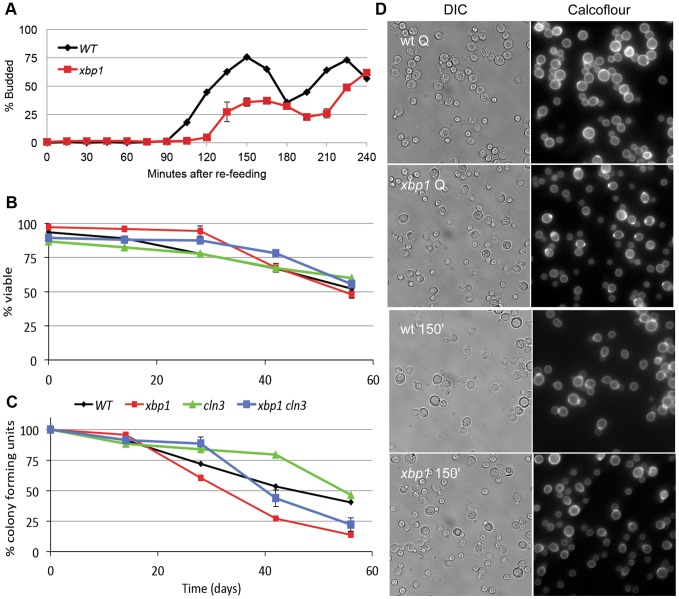
Xbp1 is important for maintaining a reversible quiescent state. (A) Percent budding as a function of time as purified Q cells are returned to fresh YEPD media and re-enter the cell cycle. (B) Long term viability and (C) colony formation of purified Q cells over 8 weeks of incubation in water. (D) Samples were taken from BY6500 wild type (WT) and *xbp1* Q cells and 150 minutes after those Q cells were re-fed. Differential image contrast (DIC) and calcofluor-stained bud scars show the budded and unbudded populations. Relevant genotypes indicated (BY6500 wild type, BY6602 *xbp1*, BY6873 *cln3*, BY7131 *cln3xbp1*.).

The high level of induction of *XBP1* suggests that it may be a major regulator during post-diauxic growth and in Q cells. Two other key properties of Q cells are their ability to rapidly reverse their arrest upon re-feeding, and their longevity during prolonged intervals of arrest. Xbp1 Q cells are defective in both of these processes. Figure 4A shows the recovery cycle of wild type and *xbp1* Q cells upon re-feeding. Wild type Q cells have a 90 minute delay, followed by a highly synchronous cell cycle as monitored by budding. *xbp1* Q cells initiate budding 30 minutes later and only about half the cells participate. The very small *xbp1* Q cells show no indication of budding at the 150 minute time point (Figure 4D.) These small cells initiate budding two hours after wild type Q cells begin to bud.

Q cell longevity is also compromised by *xbp1*. Figure 4B shows that *xbp1* Q cells, suspended in water, retain wild type viability for at least 8 weeks, as assayed by vital dye exclusion. Q cells do not acidify the water over this time course, indicating that they are in a fundamentally different state than stationary phase cultures [25]. However, *xbp1* Q cells lose the ability to form colonies more rapidly than wild type Q cells, indicating that they cannot maintain a reversible quiescent state (Figure 4C). After six weeks, 75% of *xbp1* Q cells are viable, but only one-third of those can re-enter the cell cycle and form a colony. Interestingly, all of the viable *cln3* Q cells can return to the cell cycle at this time point. This irreversible non-dividing state or senescence exhibited by *xbp1* Q cells can be delayed, but it is not suppressed by deleting *CLN3*. This indicates that the premature senescence of *xbp1* is not a Cln3-dependent phenotype. We conclude that Xbp1 also targets genes that influence the recovery and longevity of Q cells.

### Xbp1 is a global repressor during the transition to quiescence

To see if Xbp1 performs a broader repressive function during the transition to quiescence, we looked for transcripts that are repressed when *XBP1* is induced. *XBP1* mRNA undergoes dramatic oscillations in cells that are synchronized to undergo metabolic and cell cycle oscillations by glucose limitation [31]. Xbp1's known targets (*CLN3*, *CYS3*, *CLN1* and *CLB2*) also display metabolic oscillations, and peak out of phase with Xbp1. Using microarray and motif search tools [32]–[34], we identified ten transcripts that undergo metabolic oscillations out of phase with Xbp1 and that contain Xbp1 binding sites in their promoters. We verified that *PIS1*, *DOG2*, and *CDC10* are bound *in vivo* by Xbp1 after the DS, just like *CLN3* (Figure 3A). We then identified 100 transcripts whose profiles in the metabolic oscillation data set were most closely correlated with the average profile of *CLN3*, *DOG2* and *PIS1* (Supplementary Figure S1) [35]. Among those 100 genes, 54 contained Xbp1 binding sites (CTCGAG/A [14]) within 800 base pairs of their translational start sites. Three of these genes encode transcription factors (*RCS1/AFT1*, *RFX1* and *NRG2*), which we also verified to be *in vivo* binding sites for Xbp1 by chromatin immunoprecipitation (Figure 3A).

**Figure 5:**
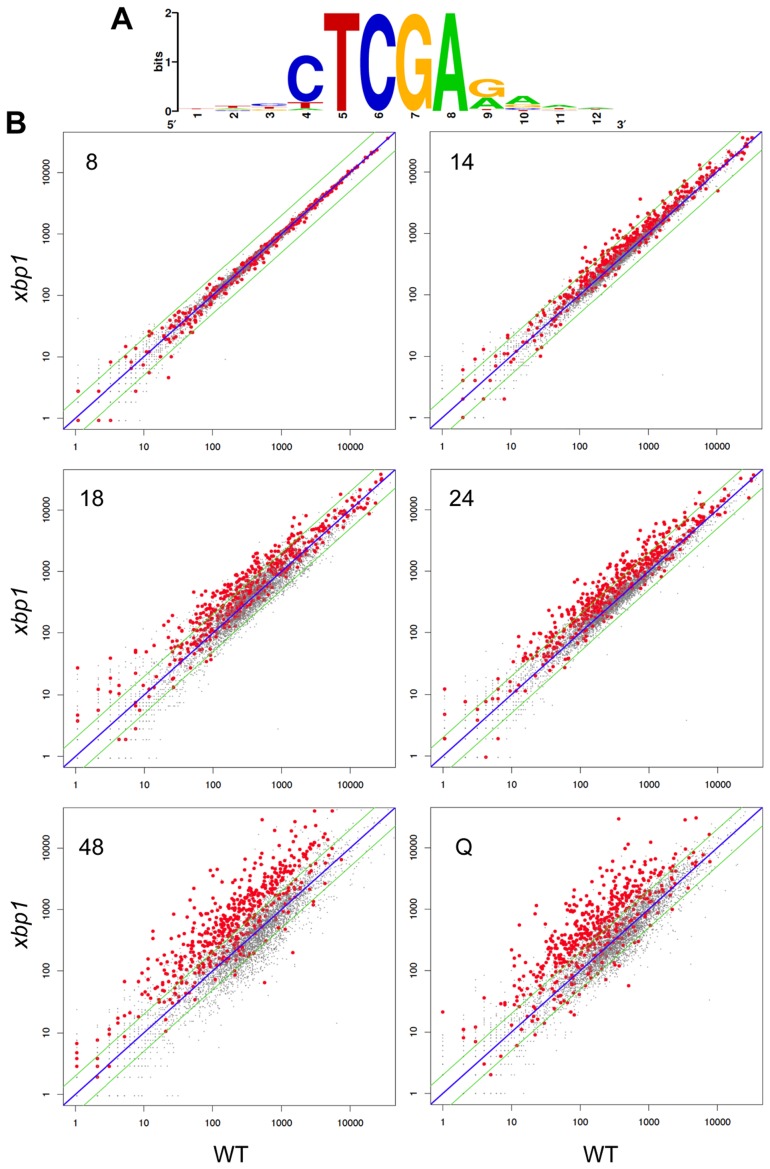
Genes with Xbp1 binding sites in their promoters are repressed after the diauxic shift. (A) Consensus Xbp1 binding site generated from the 520 Xbp1 repressed genes identified in this study. (B) Dot plot of Next-Generation RNA sequencing data for all polyadenylated transcripts from wild type (X-axis) and *xbp1* (Y-axis) cells grown to log phase (8 hours), after the DS (14 hours), after 18 or 24 hours of growth into stationary phase, and from purified Q cells. 520 Xbp1-repressed genes with Xbp1 binding sites in their promoters are highlighted in red. All other transcripts are plotted in grey.

To show that the repression of these transcripts is Xbp1-mediated and to identify other targets, we used our Next-Generation RNA sequencing [16] data to compare transcript levels of genes from wild type and *xbp1* cells as they transit from log phase to stationary phase. *CLN3* (Figure 3C), and all 54 of the transcripts we identified as having Xbp1 binding sites, were derepressed in one or more of the post-DS time points in the *xbp1* mutant. We then identified over 800 transcripts (Supplementary Table S1) that are repressed by Xbp1, three-fold or more, in at least one of the post-DS time points. More than half (520) of these genes contained Xbp1 binding sites within the 800 base pairs upstream of their coding sequences. Figure 5A shows the consensus Xbp1 binding site derived from these 520 derepressed transcripts. We will refer to these 520 genes as direct targets of Xbp1. Figure 5B shows a dot plot comparison of all transcript levels in *xbp1* and wild type cells. Direct Xbp1 targets (red dots) are not significantly affected by the absence of Xbp1 during log phase (8 hours). A few transcripts begin to rise in the *xbp1* cells at the DS (14 hours), and this trend continues throughout the time course and in purified Q cells. Very few direct targets are down-regulated. This is consistent with our previous findings that Xbp1 functions as a repressor [14], [15], and expands its role as a global repressor specifically during post diauxic growth and quiescence.

**Figure 6:**
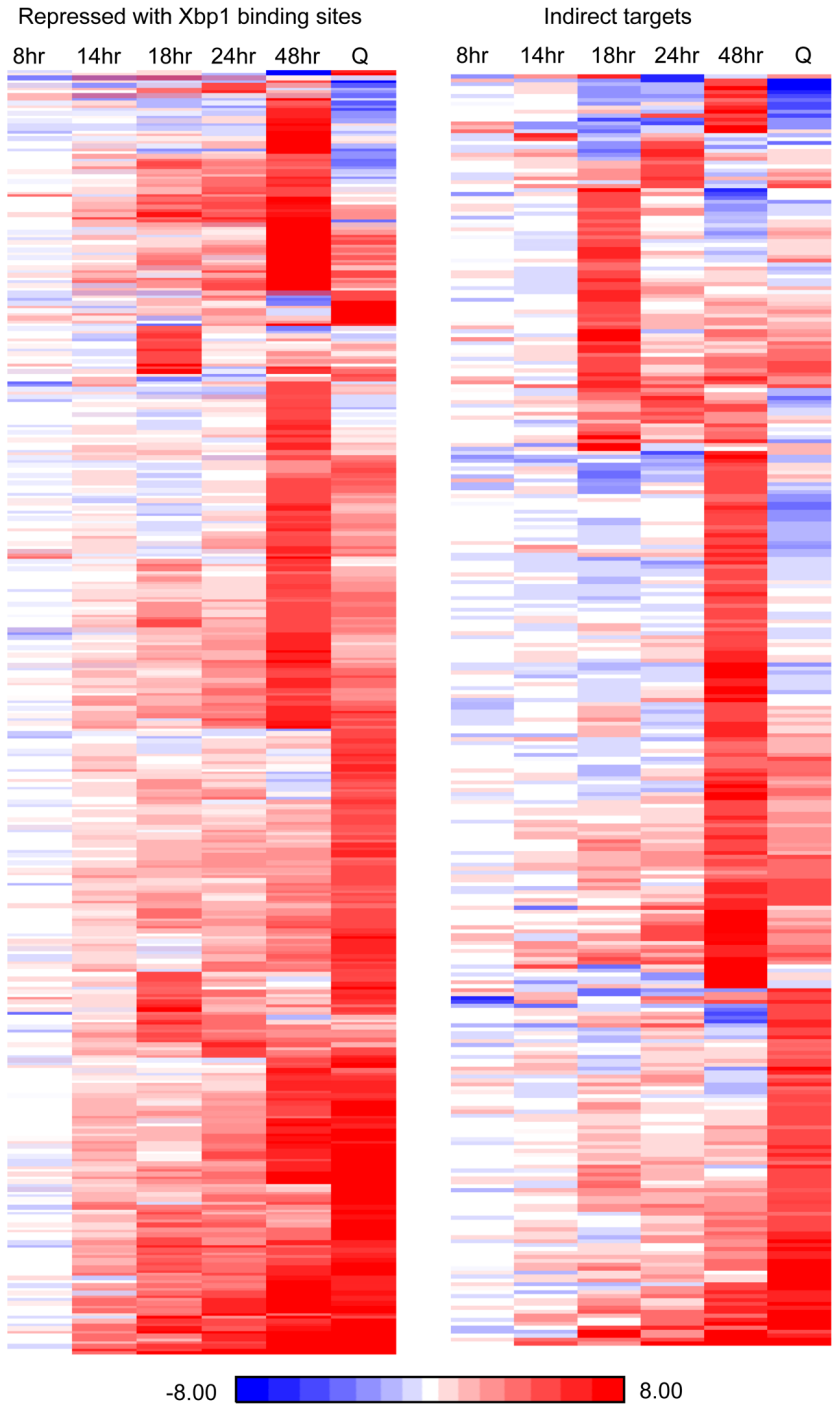
Fifteen percent of yeast transcripts are derepressed three-fold or more in *xbp1* cells during post-diauxic growth. (Left panel) derepressed genes with, or (right panel) without Xbp1 binding sites are shown after, 8, 14, 24, or 48 hours of growth and from purified Q cells (Q). RNA Next Generation sequence data are displayed as a ratio of BY6602 *xbp1*/BY6500 wild type.

Xbp1 expression is induced at 14 hours and remains high across this time course (Figure 3B), but both the levels and the timing of transcription of its targets vary widely. Figure 6 shows the transcript levels of the direct and indirect targets of Xbp1 that are elevated three-fold or greater during post-diauxic growth. Forty of these transcripts are elevated at least sixteen-fold. However, most reach their peak during a specific interval, which varies for each target gene. We speculate that this variation is due to differences in activation. If Xbp1 serves solely as a repressor, the expression of each one of its target genes would still depend on the expression and stability of its activator(s).

To look more closely at all Xbp1-mediated repression, we identified transcripts that are derepressed three-fold or greater at each time point (Table 1). Significant derepression is observed after 18 hours. The majority of these Xbp1-repressed transcripts are involved in biological regulation (p value 10–9). One third are localized to the cell periphery, but only nine are classified as cell wall proteins. 23 are localized to sites of polarized cell growth [36]. Several of these genes are involved in bud site selection or are components of the Cdc42-mediated cell polarization pathway. Components of the septin ring, which separates mother from daughter [37], the cohesion complex, which holds sister chromatids together, and components that facilitate chromosome segregation [38] are repressed by Xbp1 at 18 hours. Two cyclins (*CLN3* and *CLN1*) that drive the G1 to S transition [6] and are known Xbp1 targets [14], [15] are elevated at this time point. Regulators of transcription are also affected. Among these are transcription factors that promote the G1 to S transition (*SWI6* [39]), the S to G2/M transition (*NDD1* [40], and others that induce alternative cell fates: filamentation (*MSS11* and *MGA1* [41]), and meiosis (*IME1* [42]). Hence, Xbp1 promotes quiescence by repressing multiple targets involved in mitotic growth and by preventing cells from adopting other developmental fates.

**Table 1:** All targets of Xbp1-mediated repression.

**Derepressed at 18 hours (118 known genes)**												
cell periphery (10-10) *sites of polarized growth (10-10)												
MUC1*	*SCD5*	*DAN4*	ENT2*	BNI4*	FKS1*	HSL1*	*ENA1*	RAX2*	*YPS6*	GIC2*	*FRE5*	MSB2*
*LSB1*	*PKH2*	*HPF1*	*FIG2*	*HXT16*	SKG3*	*SLM1*	YAP1801*	*FLO5*	KCC4*	MSB1*	*PRY3*	*PMA1*
AXL2*	*FKS3*	*CAN1*	HKR1*	YAP1802*	*GPB1*	*PMA2*	*ALR2*	*NCP1*	*DNF2*	SRL1*		
PAM1*	ZDS1*	SWE1*	WSC3*	FIR1*	SSD1*							
cell division (10-06) *mitotic cell cycle (10-05)												
IRR1*	NKP2*	*KCC4*	*BNI4*	SSD1*	HSL1*	*AXL2*	*CLN1*	BFA1*	*NET1*	*RAX2*	SMC1*	GIC2*
*HKR1*	KIP1*	CLN3*	CTF3*	SWE1*	PDS5*	APC1*	CLA4*					
NDD1*	SAP4*	NET1*	ZDS1*	SWI6*	AFT1*	MLP2*	GAC1*					
regulation of gene expression (10-05) *transcription (0.00164)												
RTG1*	IXR1*	KNS1*	*SSD1*	IES1*	MSS11*	SNF5*	NET1*	ZDS1*	RLM1*	TRA1*	GAT3*	SWI6*
*NGR1*	*PKH2*	RSC30*	*MPT5*	MIG1*	MLP2*	GAC1*	*PET122*	NDD1*	RCO1*	SWC7*	ROX1*	MGA1*
SRB8*	DPB2*	GAL11*	IME1*	CLN3*	YAP7*	SNT1*	AFT1*	*CLA4*				
regulation of metabolism (10-5, 40 genes)												
**Derepressed at 24 hours (65 known genes)**												
cell division (10-11) *cytokinesis (10-10)												
HOF1*	*CLB4*	*KIP2*	*NKP2*	CTS1*	*CLN1*	DSE4*	BUD4*	CHS2*	RAX2*	GIC2*	*CLB2*	SCW11*
*CDC5*	CDC3*	IPL1*	SUN4*	*PCL9*	EGT2*	DSE1*	DSE2*					
cell periphery (10-09) *cell wall ( 10-18)												
*HOF1*	CCW12*	GAS3*	SCW4*	*SUR7*	CTS1*	GAS5*	EXG2*	SCW10*	*PMA1*	TOS1*	DSE4*	*BUD4*
*RAX2*	*TPO2*	CWP1*	*GIC2*	SCW11*	GAS1*	CDC3*	*LSB1*	SUN4*	CIS3*	EGT2*	DSE2*	SRL1*
regulation of cyclin-dependent kinase activity (0.02988)												
*PCL9*	*CLB4*	*CLB2*	*CLN1*									
reproduction (0.0449)												
*BUD4*	*CWP1*	*GIC2*	*CDC5*	*CCW12*	*IPL1*	*SPO16*	*NKP2*	*SCW4*	*SWI5*	*SUR7*	*HO*	*SCW10*
*DSE1*												
transcription (1)												
*YAP7*	*SWI5*	*PCL9*	*DPB2*	*NRM1*	*GAS1*							
**Derepressed at 48 hours (394 known genes)**												
oxidation-reduction process (10-17, 82 genes)												
phosphorus metabolism (10-11, 115 genes)												
organonitrogen compound metabolism (10-10, 102 genes)												
cell wall organization or biogenesis (10-11)												
*SIM1*	*SSP1*	*CRH1*	*BCH1*	*KRE9*	*GFA1*	*YGP1*	*PSA1*	*ECM33*	*YPS6*	*UTH1*	*PIR1*	*CIS3*
*KTR2*	*PIR3*	*SED1*	*CWP2*	*CCW12*	*FLC1*	*HSP150*	*BIT61*	*CCW14*	*EXG2*	*YPS3*	*YPS1*	*SPO73*
*MID2*	*CWP1*	*PST1*	*EXG1*	*CWH43*	*GAS1*	*BGL2*	*OSW2*	*SKG1*	*TEP1*	*PRS4*	*SPS100*	*RCR1*
*SRL1*												
cell cycle (1)												
*SSP1*	*TUB1*	*RCK2*	*SAP4*	*HAC1*	*CDC10*	*RSR1*	*CLN2*	*PSA1*	*MCM5*	*BFR1*	*UTH1*	*GIC2*
*PCL2*	*DBF2*	*PCL1*	*MMR1*	*GIN4*	*SPO16*	*PCL9*	*RME1*	*MER1*	*PCL5*	*SCP160*	*KIP2*	*REC114*
*ASH1*	*SPC25*	*FIN1*	*TVP38*	*MEI4*	*FUS3*	*TUB3*	*SMC2*	*CLN1*	*AXL2*	*ALK2*	*CHS2*	*CLB2*
*TUB2*	*CDC3*	*SWI5*	*MCM3*	*TEM1*	*CLA4*							
transcription (1)												
*MCM5*	*UTH1*	*CBF1*	*ABF1*	*EMI2*	*YAP7*	*ASH1*	*SWI5*	*CSE2*	*HAC1*	*RME1*	*MAC1*	*HDA1*
*SMP1*	*SCP160*	*VPS36*	*SPT4*	*REG2*	*UTH1*	*HIF1*	*NPL3*	*NPT1*	*GAS1*	*MCM3*	*NRG2*	
**Derepressed in Q cells (360 known genes)**												
oxidation-reduction process (10-5, 50 genes)												
small molecule metabolism (.0019, 74 genes)												
carbohydrate metabolism (.0109, 38 genes)												
transmembrane transport ( 0.0004)												
*LHS1*	*OLI1*	*PAM18*	*MIR1*	*AGP2*	*ECM22*	*MUP3*	*VMA9*	*VBA3*	*NPR1*	*STV1*	*MEP2*	*ATP8*
*KAR2*	*ATP6*	*GAP1*	*HXT9*	*SIT1*	*GGC1*	*PHO90*	*THI73*	*MPH2*	*SIL1*	*FMP13*	*FCY2*	*HXT3*
*AGC1*	*QDR2*	*ECM27*	*TPO2*	*SSA2*	*HXT6*	*ATP20*	*MDL1*	*HXT15*	*UGA4*	*AAC3*	*AGP3*	*LEU5*
*ALR2*	*ENA2*	*HXT7*										
cell cycle (1)												
*CLB4*	*CDC15*	*CNN1*	*TUB1*	*SAP4*	*HAC1*	*CDC10*	*RSR1*	*CDC27*	*CEF1*	*APC5*	*CDC48*	*BFA1*
*BUD3*	*UTH1*	*BFR1*	*KIN4*	*GIC2*	*SWI6*	*SUN4*	*APC1*	*SAE2*	*BIK1*	*SIT4*	*NKP2*	*TVP38*
*MEI4*	*SMC2*	*SML1*	*DPB2*	*ALK2*	*IME1*	*KIP1*	*CDC3*	*IPL1*	*CDC39*	*SNT1*	*MPC54*	*AFT1*
*LDB18*												
transcription (1)												
*STD1*	*HMLα1*	*MET28*	*ECM22*	*GAL4*	*HAC1*	*PAU8*	*YRM1*	*SMP1*	*SPT4*	*DAL80*	*IME1*	*SWI6*
*MATα1*	*YAP7*	*GSM1*	*CDC39*	*MAC1*	*HMLα2*	*MATα2*	*KAE1*	*AFT1*	*HDA1*	*MIG1*		

Organized by GO categories. Corrected P value for enrichment and number of genes are shown in parentheses.

Genes associated with subcategories are noted with asterisks.

After 24 hours of growth, 65 known genes are derepressed in the absence of Xbp1. At this time point, cell wall proteins are highly enriched. These include most of the daughter-specific genes [43]. Six gluconases and the chitinase Cts1, which are responsible for degrading the cell wall and chitin ring between mother and daughter to achieve cell separation [44] are targeted. In addition, cell division and specifically cytokinesis targets are highly enriched. Three late cycle cyclins (*CLB4*, *CLB2* [45] and *PCL9* [46]) are also targeted.

By 48 hours, nearly 10% of all genes (515) are derepressed in the absence of Xbp1. At this time point almost half of the known targeted genes are involved in metabolism and the other large class is involved in cell wall biogenesis. 45 cell cycle genes and 25 transcription regulators are also derepressed at this time point. Only one-third of these derepressed genes are also derepressed in Q cells. Xbp1 affects a more diverse group of genes in purified Q cells. Metabolic genes are the largest class. In addition, 42 genes involved in transmembrane transport, including five glucose transporters are repressed by Xbp1 in Q cells.

We also analyzed direct and indirect targets separately. What is striking is that direct and indirect targets are largely in the same pathways. At 18 and 24 hours, mitosis, cell cycle, cell division and cytokinesis are significantly enriched classes in both direct and indirect targets (Supplementary Table S2). At 48 hours, both direct and indirect targets are highly enriched for genes involved in metabolism and cell wall organization.

The dot plots of Figure 5 show that Xbp1 primarily serves as a repressor of transcription. Transcripts whose levels are under-represented by three-fold or more in the *xbp1* mutant are rare until the 48 hour time point and in Q cells. However, at these two time points almost 500 transcripts fit this criterion. Unlike the derepressed transcripts, of which 60% are associated with Xbp1 binding sites, only one fifth of the down-regulated genes are near an Xbp1 binding site, which is about what is expected by chance. This is consistent with Xbp1 playing an indirect role at these promoters. To our surprise, there are only 14 transcripts that are under-represented both at 48 hours and in Q cells. At 48 hours, the under-represented transcripts are nearly all involved in ribosome biogenesis (90/257, p value 10–45) and nitrogen metabolism (156/257 p value 10–18). In Q cells, they are highly enriched for ribosomal proteins (47/129, p value 10–42) and genes involved in monosaccharide catabolism (16/129 p value 10–12). The ribosome biogenesis and ribosomal protein transcripts are tightly and coordinately regulated in response to nutrient conditions [47]. It is unclear how Xbp1 influences the expression of these genes. The striking lack of overlap between the transcripts that are under-represented in *xbp1* cells at 48 hours versus purified Q cells suggests that these are fundamentally different states.

## Discussion

In rich glucose-containing medium, yeast cells cease growth and division after about 48 hours due to carbon limitation. The resulting culture is a heterogeneous population of live and dead cells. Most of the daughter cells enter a quiescent state and can be purified due to their increased density [1]. Q cells develop unique characteristics including high thermo-tolerance [1] and high levels of glucose stored in the form of trehalose and glycogen [4]. The transition to quiescence does not occur when cells are abruptly deprived of glucose (Li et al, submitted), so there must be some cellular response to its waning supply that signals cells to stop proliferating, stockpile the remaining glucose and enter a quiescent state.

We are investigating the events that differentiate Q cells from nonQ cells and promote their longevity [48]. We find that G1 arrest is an early event in the transition to quiescence. There is a three-fold increase in the fraction of cells in G1 that occurs before glucose is depleted from the medium. At this point, referred to as the diauxic shift (DS), initiation of DNA synthesis is dramatically reduced and most of the cell division that occurs thereafter can be accounted for by the completion of cell cycles that were previously initiated. We have identified several key regulators that are important for achieving this arrest. We find that excess Cln3 activity, expressed from five integrated copies of the wild type *CLN3* gene, interferes with Q cell formation, and cells lacking Cln3 produce more Q cells. Cells transitioning to quiescence with excess Cln3 accumulate in G1 more slowly than wild type cells, but they eventually arrest and remain viable due to the activation of the checkpoint kinase Rad53.

Rad53 is an effector of the DNA damage and replication stress checkpoints [49]. Rad9, which is specific to the DNA damage checkpoint [29], [30], is not required for the survival of *5XCLN3* cells, so we conclude that replicative stress, not DNA damage, triggers the checkpoint during the transition to quiescence. We detect delayed G1 arrest and increased ROS accumulation as nutrients become limiting, even in wild type cells carrying *rad53-21*. This suggests that replication stress occurs and this checkpoint pathway plays a role in restricting cell cycle progression during the wild type transition to quiescence. With excess Cln3, checkpoint function becomes essential and cells lacking it fail to arrest in G1 and undergo apoptosis. Related effects have been observed with excess cyclin E, and other activated oncogenes in higher cells [50], [51] and in yeast [26], [27], [52].

Our data indicate that *CLN3* repression is mechanistically different before and after the DS, and that only its post-DS repression is Xbp1-dependent. The initial drop in *CLN3* levels and the halt to S phase that we observe at the DS are Xbp1-independent. Only after the DS, the *CLN3* promoter is bound and repressed by Xbp1. Cells lacking Xbp1 resume DNA replication and continue to divide after the DS, and this results in a significant population of very small cells. These phenotypes are Cln3-dependent. These data are consistent with Xbp1 playing a role in maintaining repression of *CLN3* and G1 arrest as cells transition from growth to quiescence. However, unlike *5XCLN3*, *xbp1* mutants are not dependent on the Rad53 replication stress checkpoint for viability. We suspect that either the timing or the extent of derepression of *CLN3* by *xbp1* could explain the Rad53-independence of these cells. A third possibility is that Rad53 acts in the same pathway and upstream of Xbp1 to restrict cell cycle progression. Rad53 has been shown to increase the level of Xbp1 in response to DNA damage [19]. These possibilities are under investigation.

Xbp1 mutant Q cells remain viable, but they are profoundly delayed in cell cycle re-entry upon re-feeding. They are also short-lived as Q cells, entering an irreversible, senescent state more rapidly than wild type. We have not identified the genes responsible for these phenotypes because our data show that Xbp1 plays a global and continuous repressive role in cells as they transition from a dividing to a non-dividing quiescent state. We have identified 520 targets of Xbp1-mediated repression that contain Xbp1 binding sites in their promoters. All seven that we tested are direct *in vivo* binding sites for Xbp1. Binding is only detected after the DS, which explains why these targets were not identified in previous studies. None of the Xbp1 targets identified by genome-wide location analysis [53] are among the 520 targets we have identified, and only 5 of the 41 transcripts reported to be affected by an *xbp1* deletion [54] are among the 822 transcripts that we find are derepressed after the DS. These differences emphasize the need to determine when a transcription factor is active and use those conditions to search for its targets. The consensus binding site we have derived from the 520 targets agrees with that which we initially identified by site selection [14] and that reported by [55].

*XBP1* mRNA oscillates dramatically in cells that are undergoing yeast metabolic and cell cycle (YMC) oscillations [31], and we identified many Xbp1 targets by looking for its binding site in transcripts that oscillate out of phase with *XBP1*. YMC oscillations are achieved by growing the cells to maximum density, starving them for glucose, then restoring a limited amount of glucose, which is immediately imported and cannot be detected in the media [31], [56]. These conditions resemble the DS and they evoke the expression of genes that are induced by glucose starvation and stress, including *XBP1*. Cell division stops and storage carbohydrates accumulate. This is the quiescence-like phase of the YMC [4]. In the subsequent phase, *XBP1* is turned off, and it's targets peak. Then, DNA replication takes place and cells divide. The striking parallels between the events associated with the transitions in and out of quiescence, and those associated with the YMC suggest that YMC oscillations may be the result of switching on and off the signal to arrest in G1 and enter quiescence. It also seems likely that the oscillation of *XBP1* expression is responsible for the subsequent YMC oscillations of its many targets. One such verified target, *CLN3*, and many other cell cycle regulated transcripts have been shown to have different peak time or multiple peaks in the synchronized cell cycles induced by the YMC protocol [57], [58] compared to that of other cell cycle synchronization studies [59]–[61]. Our finding that a global repressor of *CLN3* and 800 other transcripts is also oscillating during the YMC time course may explain some of those altered peak times.

Xbp1 negatively regulates the mRNA levels of 15% of yeast genes during post-diauxic growth. When Xbp1 was ectopically expressed during logarithmic growth, only a small number of Xbp1 targets were identified [14]. This suggests that Xbp1 may be more active in the post-diauxic state, either due to modification of Xbp1 or to the presence of co-factors that increase its activity or the accessibility of its targets. Among its many targets, Xbp1 represses the transcription of key activators of mitosis, meiosis, and filamentation. Repressing these genes may promote the quiescent state by reinforcing G1 arrest and by preventing cells from adopting alternative fates that are also triggered by nutrient limitation. Xbp1 also plays a major but complex role in the metabolic shifts that take place as cells shift from glycolysis to respiration to quiescence. Its many structural and regulatory targets involved in cell wall remodeling and cell division indicate that preventing growth is an active and continuous process in quiescent cells. Even basal expression of these genes may be deleterious to the stability of the quiescent state and/or to the orderly recovery from it.

It is striking that transcripts derepressed by *xbp1* early in the transition to quiescence are largely cell cycle and growth regulators. At 18 hours, even the gene products associated with the cell periphery are largely sensors and regulators, rather then structural proteins. This is true of both direct and indirect targets. One possible explanation is that *xbp1* mutant cells continue to divide during this interval. About 20% of Xbp1 targets are cell cycle regulated at the transcript level (data not shown). These transcripts are not made when cells stop dividing, so anything that promotes ectopic cell division would increase the transcription of these genes. However, only one-third of the 600 most cell cycle regulated transcripts [62] are elevated in the absence of Xbp1 and no particular class is enriched. If their elevated levels were due to continued cell divisions, all 600 would be elevated. We conclude that repression of these transcripts is an active Xbp1-mediated process, and the fact that half of them contain Xbp1 binding sites in their promoters is consistent with that conclusion. By 48 hours, hundreds of transcripts involved in metabolism and cell wall organization are affected. Again this is true of direct and indirect targets. At this point cell division has ceased, and transcriptional activators that promote cell division are likely to be inactive. Without these activators, loss of Xbp1-mediated repression may be of little consequence. However, house-keeping and metabolic genes may be constitutively active and require sustained repression in order to conserve resources. Our data indicate that Xbp1 provides the repression of these genes, perhaps through its recruitment of the histone deacetylase, Rpd3 [19].

We also looked for transcripts that were under-represented in the *xbp1* mutant. These were prominent only in the last two time points and they do not show any enrichment for Xbp1 binding sites. This supports the view that Xbp1 functions primarily, if not solely, as a repressor. We expect that the reduced levels of these transcripts are an indirect effect of the many perturbations that arise in *xbp1* cells where 15% of genes are expressed at a time when they should be off.

*XBP1* mRNA is among the top 1% highest level transcripts in Q cells. Xbp1 has also been shown to be translationally up-regulated in response to both glucose and amino acid starvation [63]. These observations are consistent with Xbp1 serving as a global repressor of transcription as cells respond to nutrient depletion and transition to a non-dividing quiescent state. The longevity and recovery defects we observe for *xbp1* mutant Q cells demonstrate the importance of this repression. Xbp1 shares homology within its DNA binding domain with four other *S. cerevisiae* transcription factors that specify cell fate. Swi4 and Mbp1 associate with Swi6 and serve as activators of mitotic growth [64]. Sok2 and Phd1 play opposing roles in pseudohyphal development [65]–[67]. Xbp1 plays a minor role in sporulation [15] and pseudohyphal development [68], and this work shows that it is an important global repressor during the transition to quiescence. This family of transcription factors is found only in fungi, and may be important targets for anti-fungal drugs. One of the *Candida* family members, Efg1, is critical for biofilm formation, which renders these pathogens drug-resistant [69]. We note that many Xbp1 targets are also known to affect virulence in bacterial and fungal pathogens. These include *PMT1*, *2*, and *4* [70], *ECM33* [71], *SMI1* and *FKS1* [72]. Understanding Xbp1's regulation and its role in defining the quiescent state may provide important insights with both medical and basic research implications.

## Materials and Methods

### Strains and growth conditions

The yeast strains were all derived from W303. The auxotrophic markers were corrected in all strains. The strains carrying five copies of *CLN3* were generated by integrating additional copies of *CLN3* at four different marker loci using the integrating vectors, pRS303-306 [73]. The wild type controls for these studies contain the same empty vectors integrated at the same locations. To generate the W303 prototroph, BY6500, the auxotrophic mutations were replaced with the wild-type sequence by homologous gene replacement and verified by PCR and sequencing. The checkpoint deficient *rad53-21* mutant (Allen *et al.*, 1994) was crossed into the W303 background above to generate BY6741 and subsequently crossed with the 5X*CLN3* strain to generate BY6698. *CLN3, XBP1 and RAD9* were deleted with *KanMX* as described [74].

Reproducible growth curves were obtained by patching cells from fresh plates onto YEP plus 2% glycerol and growing them overnight to eliminate petites. This patch was used to inoculate 5 ml YEPD, then a further 1/50 dilution was made and grown overnight. This culture was used to inoculate 25 ml YEPD in a 250 ml flask to an optical density (OD_600_) of 0.02 and allowed to grow at 30°C, shaking at 200 RPM. The diauxic shift was defined as the point at which no glucose was detected in the media, which was determined with glucose detection strips (GLU 300, Precision labs, Inc. West Chester, OH). Quiescent (Q) cells were purified from YEPD cultures that were seven days old using a 25 ml percoll density gradient [1] with minor modifications [48]. Q cell yield is calculated as the percentage of OD_600_ units loaded that sediment to the bottom nine ml of the gradient. Cell size and cell count was measured on a Z2 Beckman Coulter Counter. All time course data was collected in duplicate or triplicate, averaged and error bars are shown.

### Viability and reproductive capacity

Cell viability was monitored using the FungaLight Yeast Viability Kit (Molecular Probes) according to the manufacturer's protocol and the percentage of live cells was plotted over time. Reproductive capacity was assayed as the ability to resume cell division and produce colonies. Serial dilutions were plated on YEPD plates in duplicate and the percentage of colony forming units (CFU) was plotted, using the CFU from the freshly harvested Q cell sample as 100%. The FungaLight and CFU viability data are averages from at least two independent experiments.

### Cell imaging

Calcofluor staining of bud scars: Approximately 10^7^ cells were collected and mixed with Calcofluor white M2R (Fluorescent brightener 28; Sigma) at a final concentration of 100 µg/ml. Cells were incubated at room temperature for 15 min in the dark then were washed twice with H_2_O. The stained cells were examined with a Nikon Eclipse E600 microscope with a Nikon Plan Apochromat 60XA/1.40 oil immersion objective and a UV-2E/C DAPI filter (excitation at 330–380 nm). Photomicrographs of cells were taken on a Photometrics Cascade 512B camera and analyzed with MetaMorph version 6.3r2 software (Molecular Devices, Sunnyvale, CA). TUNEL Assay: Cells were fixed with 4% paraformaldehyde at room temperature for 15 min, spun down at 5000 rpm for 5 min and washed once with 0.1 M potassium phosphate 1.2 M sorbitol buffer pH 7.5. Stationary phase cells were first resuspended in 100–200 µl fresh pretreatment buffer (1 M Sorbitol, 25 mM EDTA, 50 mM DTT, pH 8), and then pelleted in a microfuge at 2000 rpm for 3 min at room temperature. These cells were resuspended in 1 M sorbitol and pelleted as before. Cell walls were digested with 50 µg/ml Zymolyase 100T in 1 M Sorbitol buffer (pH 5.8) for 10–45 min at 30°C. Cells were pelleted at 2000 rpm for 3 min, gently washed and resuspended in 15 µl potassium phosphate/sorbitol buffer, transferred to 0.1% polylysine-coated wells of an eight well microscope slide and allowed to settle for 20 min at room temperature. The slide was washed twice with phosphate-buffered saline (PBS). Each well was incubated with 40 µl fresh permeabilization solution (0.1% Triton X-100 in a 0.1% sodium citrate solution) for 2 min on ice, then rinsed with PBS buffer. 15 µl TUNEL reaction mixture (In Situ Cell Death Detection Kit, AP, Roche) was added to each well, slides were covered and incubated for 60 min at 37°C, then rinsed twice with PBS. Cells were observed under the microscope with an FITC filter (excitation at 460–500 nm). 100–200 cells per sample were evaluated.

### Flow cytometry

For flow cytometry, cells were fixed in 70% ethanol for two hours or overnight, washed once with water, then resuspended in .5 ml 50 mM Tris-HCl (pH 8.0) containing 0.2 mg/ml RNAse A and incubated at 37°C for four hours. These cells were spun down, resuspended in .5 ml 50 mM Tris-HCl (pH 7.5) containing 2 mg/ml Proteinase K, and incubated at 50°C for one hour. They were then spun down again and resuspended in .5 ml 50 mM Tris-HCl (pH 7.5) and stored at 4°C. Before analysis, they were sonicated, pelleted, and resuspended in .5 ml I.0 µM Sytox Green (Invitrogen). Percent of cells in G1, S or G2/M phase of the cell cycle were quantified with FlowJo V9.

For ROS assays, approximately 1×10^6^ cells were pelleted, gently washed with PBS, then resuspended in 1 mL PBS. 2.5 µL of a 10 mM carboxy-H2DCFDA (Invitrogen) stock solution was added and the cells were incubated for 30 minutes at 37°C. Cells were washed twice with PBS, resuspended in 1 mL 50 mM Tris-HCl pH 7.5. Cultures were then sonicated and 30,000 cells per sample were collected on a Fluorescence Activated Cell Sorter FACScan cytometer (BD Biosciences, San Jose, CA) and analyzed using Cell Quest software. FACS parameters were set at excitation and emission settings of 495 nm and 529 nm (filter FL-1), respectively. Average from two experiments is reported.

### RNA measurements

To generate enough cells for RNA measurements during growth from log phase to stationary phase, 5 OD_600_ of cells were collected every 10 minutes, washed with RNA buffer (50 mM Tris•HCl pH 7.4, 100 mM NaCl, 10 mM EDTA) and frozen for later RNA purification. The levels of *CLN3* and *ACT1* mRNA were monitored by an S1 nuclease protection assay as previously described [61]. *CLN3* and *ACT1* transcript levels were measured in each sample of wild type and *5XCLN3* cells. The *ACT1*, though not invariant, was not affected by excess CLN3 so it could be used to normalize the RNA levels between the two strains.

Next-Generation RNA sequencing was carried out with RNA prepared as above from log phase cells, purified Q cells, and cells grown in YEPD to log phase (8 hours) the DS (14 hours), then 18, 24 and 48 hours. mRNA expression levels following polyA selection were assayed using the HiSeq 2000 next generation sequencing system from Illumina [16], with RNA libraries prepared according to the manufacturer's instructions. FASTQ sequence output files were generated, demultiplexed by the Illumina CASAVA software package and filtered to remove sequences with low read quality. Nucleotide fragments were paired-end sequenced.

The W303 reference genome in FASTA format and gene annotations in GFF were obtained from the Wellcome Trust Sanger Institute's SGRP group. Sequences from each read were mapped to the *Saccharomyces cerevisiae* W303 reference genome using the Tophat application, a fast splice junction mapper for RNA-Seq reads [75]. Representation of RNA from annotated genes were assessed using HTSeq, a Python package developed by Simon Anders at EMBL Heidelberg, with quantitative expression calculated proportional to the number of reads per length of the modeled exon (MRPKBME). Finally, differential gene representation between treatments were assessed using the R/Bioconductor package DESeq [76]. These data for differentially expressed genes are provided as Supplementary Table S3. The demultiplexed FASTQ files have been submitted to the National Center for Biotechnology Information Sequence Read Archive and are available there as accession SRA098245.

### Chromatin immunoprecipitation

Cells carrying Xbp1 tagged with a Tandem Affinity Purification (TAP) tag [77] or a non-tagged Xbp1 were collected from log phase cultures and cultures that had been growing for 24 hours into stationary phase. Proteins were cross linked to DNA as described [78] and IgG agarose beads (Sigma A2909) were used to pull down *in vivo* binding sites. PCR primers used to amplify potential targets as well as an unregulated DNA (IRV) are provided as Supplementary Table S4.

## Supporting Information

Figure S1 XBP1 transcript oscillates out of phase with its targets in cells synchronized with limiting glucose. (A) Metabolic oscillations of mRNA for *XBP1* (green), and three of its direct targets: *PIS1* (blue), *CLN3* (red) and *DOG2* (purple), reproduced from the Periodic Transcript Server [35]. (B) 50 other transcripts whose profiles were most closely correlated with the average profile of *CLN3*, *DOG2* and *PIS1* in the metabolic oscillation data set [31].
(TIF)

Table S1 All transcripts derepressed three-fold or more by *xbp1*. Open reading frame number and classification, gene name and gene description are provided. TRUE indicates genes with Xbp1 binding sites within 800 base pairs of translational start sites. FALSE indicates indirect targets.
(XLSX)

Table S2 Xbp1 repressed genes after different intervals of growth. (A) Targets containing Xbp1 binding sites or (B) that lack Xbp1 binding sites that are listed based on when they are derepressed, that is after 18, 24, 48 hours of growth or in purified Q cells. Genes are organized by gene ontology (GO) process. Corrected P value and the number of genes in each class are shown in parentheses.
(DOC)

Table S3 Next Generation RNA sequence data for Xbp1 targets. Data for all transcripts derepressed three-fold or more at one or more time point are provided. Open reading frame number and classification, and gene name are provided. TRUE indicates genes with Xbp1 binding sites within 800 base pairs of translational start sites. FALSE indicates indirect targets. For each target gene at each time point, the data for *xbp1* and wild type cells are normalized and provided as a log base 2 value for xbp1/wild type. Rank is provided as an indicator of the likelihood that the genes are differentially expressed in the two strains at each time.
(XLSX)

Table S4 PCR primers used in chromatin immunoprecipitations.
(DOCX)
