# Supplementary figures and images for: Xbp1 Directs Global Repression of Budding Yeast Transcription during the Transition to Quiescence and Is Important for the Longevity and Reversibility of the Quiescent State
Source: PLoS Genet. 2013 Oct 31;9(10):e1003854. doi: 10.1371/journal.pgen.1003854 (PMC3814307; doi:10.1371/journal.pgen.1003854)

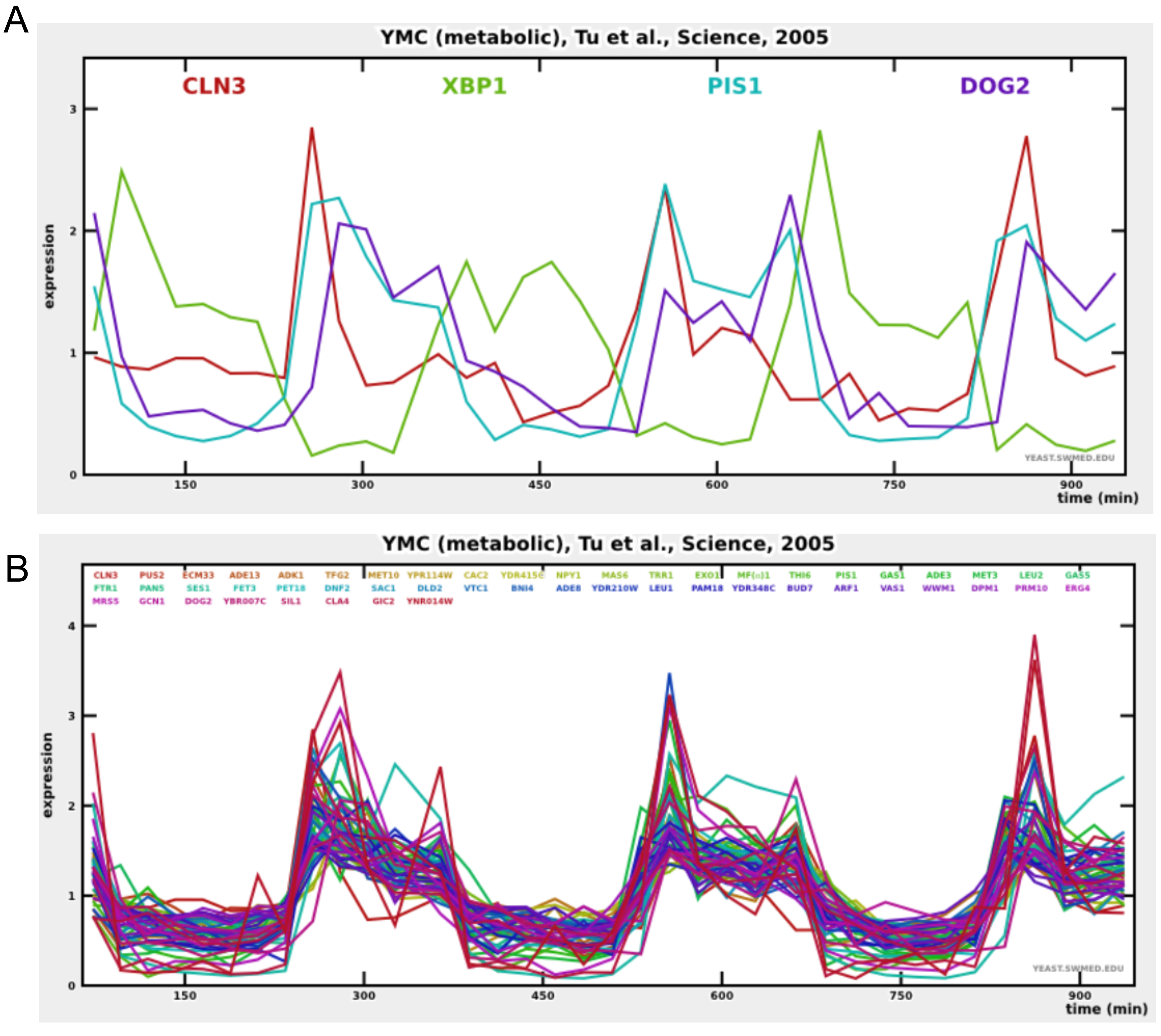

Supplement: Figure S1 — XBP1 transcript oscillates out of phase with its targets in cells synchronized with limiting glucose. (A) Metabolic oscillations of mRNA for XBP1 (green), and three of its direct targets: PIS1 (blue), CLN3 (red) and DOG2 (purple), reproduced from the Periodic Transcript Server [35]. (B) 50 other transcripts whose profiles were most closely correlated with the average profile of CLN3, DOG2 and PIS1 in the metabolic oscillation data set [31]. (TIF) [file pgen.1003854.s001.tif]
