# Supplementary material for: Xbp1 Directs Global Repression of Budding Yeast Transcription during the Transition to Quiescence and Is Important for the Longevity and Reversibility of the Quiescent State
Source: PLoS Genet. 2013 Oct 31;9(10):e1003854. doi: 10.1371/journal.pgen.1003854 (PMC3814307; doi:10.1371/journal.pgen.1003854)
Supplement: Table S2 — Xbp1 repressed genes after different intervals of growth. (A) Targets containing Xbp1 binding sites or (B) that lack Xbp1 binding sites that are listed based on when they are derepressed, that is after 18, 24, 48 hours of growth or in purified Q cells. Genes are organized by gene ontology (GO) process. Corrected P value and the number of genes in each class are shown in parentheses. (DOC) [file pgen.1003854.s003.doc]

| SupplementaryTable S2A: Xbp1-repressed genes containing Xbp1 binding sites | | | | | | | | | | | |  |
| --- | --- | --- | --- | --- | --- | --- | --- | --- | --- | --- | --- | --- |
| Derepressed at 18 hours (69 known genes) | | | | |  |  |  |  |  |  |  |  |
| regulation of cell shape (10-4) | | | |  |  |  |  |  |  |  |  |  |
| *FKS1* | *GIC2* | *FIG2* | *YPK2* | *FKS3* |  |  |  |  |  |  |  |  |
| (1->3)-beta-D-glucan biosynthetic process (0.0014) | | | | | |  |  |  |  |  |  |  |
| *FKS1* | *HKR1* | *FKS3* |  |  |  |  |  |  |  |  |  |  |
| regulation of cell cycle (0.0145) | | | |  |  |  |  |  |  |  |  |  |
| *ZDS1* | *GIC2* | *IME1* | *SWI6* | *CLN3* | *SNT1* | *HSL1* | *CLN1* | *CLA4* |  |  |  |  |
| establishment of cell polarity (0.0497) | | | | |  |  |  |  |  |  |  |  |
| *RAX2* | *ZDS1* | *GIC2* | *HKR1* | *DNF2* | *AXL2* | *CLA4* |  |  |  |  |  |  |
| cell cycle (0.051) | |  |  |  |  |  |  |  |  |  |  |  |
| *SAE2* | *NKP2* | *SAP4* | *HSL1* | *AXL2* | *CLN1* | *RAX2* | *GIC2* | *ZDS1* | *HKR1* | *KIP1* | *IME1* | *CLN3* |
| *AFT1* | *CLA4* | *SWI6* | *SNT1* |  |  |  |  |  |  |  |  |  |
| transcription (0.68) | | |  |  |  |  |  |  |  |  |  |  |
| *KNS1* | *NAB3* | *SWC7* | *IES1* | *ROX1* | *MGA1* | *ZDS1* | *RLM1* | *GAT3* | *IME1* | *CLN3* | *SWI6* | *YAP7* |
| *MIG1* | *SNT1* | *AFT1* |  |  |  |  |  |  |  |  |  |  |
| Derepressed at 24 hours (50 known genes) | | | | |  |  |  |  |  |  |  |  |
| cell division (10-8) | | |  |  |  |  |  |  |  |  |  |  |
| *HOF1* | *CLB4* | *KIP2* | *NKP2* | *CTS1* | *CLN1* | *DSE4* | *BUD4* | *CHS2* | *RAX2* | *GIC2* | *CLB2* | *SCW11* |
| *DSE1* | *CDC3* | *SUN4* |  |  |  |  |  |  |  |  |  |  |
| cytokinesis (10-8) | | |  |  |  |  |  |  |  |  |  |  |
| *BUD4* | *HOF1* | *RAX2* | *CHS2* | *SCW11* | *GIC2* | *CDC3* | *SUN4* | *CTS1* | *DSE4* | *DSE1* |  |  |
| carbohydrate metabolic process (0.0077) | | | | |  |  |  |  |  |  |  |  |
| *GAS3* | *GAS1* | *GAS5* | *SCW4* | *SCW10* | *SCW11* | *GDB1* | *DOG2* | *CTS1* | *EXG2* | *TKL1* |  |  |
| cell wall organization or biogenesis (0.077) | | | | |  |  |  |  |  |  |  |  |
| *CIS3* | *CWP1* | *CCW12* | *EXG2* | *GAS1* | *DSE4* | *SRL1* |  |  |  |  |  |  |
| transcription (1) | |  |  |  |  |  |  |  |  |  |  |  |
| *YAP7* | *GAS1* |  |  |  |  |  |  |  |  |  |  |  |
| Derepressed at 48 hours (262 known genes) | | | | |  |  |  |  |  |  |  |  |
| oxidation-reduction process (10-10, 50 genes) | | | | | |  |  |  |  |  |  |  |
| organonitrogen metabolic process (10-8, 64 genes) | | | | | |  |  |  |  |  |  |  |
| phosphorus metabolic process (10-8, 68 genes) | | | | | |  |  |  |  |  |  |  |
| cell wall organization or biogenesis (10-6) | | | | |  |  |  |  |  |  |  |  |
| *SED1* | *SIM1* | *CCW12* | *FLC1* | *HSP150* | *CRH1* | *KRE9* | *GFA1* | *CCW14* | *EXG2* | *YGP1* | *PSA1* | *MID2* |
| *CWH43* | *GAS1* | *PIR1* | *SKG1* | *TEP1* | *CIS3* | *PRS4* | *RCR1* | *SRL1* | *CWP1* | *EXG1* | *UTH1* | *YPS6* |
| septin ring organization (0.0237) | | | |  |  |  |  |  |  |  |  |  |
| *PCL2* | *CDC10* | *PCL1* | *CDC3* | *GIN4* | *CLA4* |  |  |  |  |  |  |  |
| conjugation (0.4839) | | |  |  |  |  |  |  |  |  |  |  |
| *MID2* | *RRI2* | *ASG7* | *AGA2* | *CCW12* | *TUB1* | *SCW4* | *FUS3* | *FIG2* | *SCW10* | *TUB3* | *BAR1* |  |
| cell division (0.5018) | | |  |  |  |  |  |  |  |  |  |  |
| *KIP2* | *SKM1* | *SPC25* | *FUS3* | *CDC10* | *RSR1* | *SMC2* | *AXL2* | *CLN1* | *UTH1* | *CHS2* | *CLB2* | *GIC2* |
| *GIN4* | *TEM1* | *RME1* | *CHS1* | *CLA4* | *PCL5* | *PCL1* | *MMR1* | *PCL2* | *CDC3* |  |  |  |
| regulation of transcription (1) | | | |  |  |  |  |  |  |  |  |  |
| *NRG2* | *YAP7* | *ASH1* | *HAC1* | *TEA1* | *RME1* | *MAC1* | *MAL13* | *HDA1* | *SMP1* | *REG2* | *SPT4* |  |
| Derepressed in Q cells (220 known genes) | | | | |  |  |  |  |  |  |  |  |
| cellular process (0.0006, 190 genes) | | | | |  |  |  |  |  |  |  |  |
| carbohydrate metabolic process (0.0041) | | | | |  |  |  |  |  |  |  |  |
| *DOG1* | *MAL12* | *GAS3* | *PMT5* | *ALG3* | *TKL1* | *SCW4* | *KRE9* | *DOG2* | *GFA1* | *GAS5* | *TAL1* | *SOL3* |
| *GRE3* | *PMT1* | *PMT2* | *PFK27* | *FKS3* | *CHS3* | *GAS1* | *GDB1* | *SEC59* | *MIG1* | *STT3* | *MNN4* | *SCW10* |
| *SGA1* | *FSP2* |  |  |  |  |  |  |  |  |  |  |  |
| response to topologically incorrect protein (0.005) | | | | | |  |  |  |  |  |  |  |
| *DFM1* | *SCJ1* | *HAC1* | *LHS1* | *KAR2* | *PMT1* | *PMT2* | *CDC48* | |  |  |  |  |
| regulation of transcription (1) | | | |  |  |  |  |  |  |  |  |  |
| *SKO1* | *IME1* | *SWI6* | *YAP7* | *HAC1* | *MAC1* | *KAE1* | *AFT1* | *HDA1* | *MIG1* | *SMP1* | *SPT4* |  |
|  |  |  |  |  |  |  |  |  |  |  |  |  |
| Supplementary Table S2B: Indirect targets | | | | | |  |  |  |  |  |  |  |
| Derepressed at 18 hours (51 known genes) | | | | |  |  |  |  |  |  |  |  |
| mitosis (10-5) | |  |  |  |  |  |  |  |  |  |  |  |
| *BFA1* | *NET1* | *IRR1* | *SMC1* | *CTF3* | *SSD1* | *SWE1* | *PDS5* | *MLP2* | *GAC1* | *APC1* |  |  |
| organelle organization (10-4) | | | |  |  |  |  |  |  |  |  |  |
| *IRR1* | *SLM1* | *YAP1801* | *KCC4* | *RCO1* | *SSD1* | *SNF5* | *BFA1* | *NET1* | *DPB2* | *TRA1* | *SMC1* | *APC1* |
| *DEF1* | *NGR1* | *ATP10* | *YRF1-2* | *CTF3* | *YRF1-4* | *RSC30* | *SWE1* | *PDS5* | *SEC31* | *MLP2* | *GAC1* |  |
| regulation of gene expression (0.0026) | | | | |  |  |  |  |  |  |  |  |
| *RTG1* | *IXR1* | *NDD1* | *RCO1* | *SSD1* | *MSS11* | *SNF5* | *NET1* | *GAL11* | *SRB8* | *DPB2* | *TRA1* | *NGR1* |
| *PKH2* | *RSC30* | *MPT5* | *GAC1* | *MLP2* |  |  |  |  |  |  |  |  |
| Derepressed at 24 hours (15 known genes) | | | | |  |  |  |  |  |  |  |  |
| cell cycle (.0025) (*M phase .0936) | | | |  |  |  |  |  |  |  |  |  |
| *DPB2* | *CDC5** | *IPL1** | *SPO16** | *SWI5** | *PCL9* | *NRM1* | *EGT2* |  |  |  |  |  |
| regulation of transcription involved in G1 (0.0444) | | | | | |  |  |  |  |  |  |  |
| *SWI5* | *NRM1* |  |  |  |  |  |  |  |  |  |  |  |
| Derepressed at 48 hours (132 known genes) | | | | |  |  |  |  |  |  |  |  |
| phosphorus metabolic process (10-7, 41 genes) | | | | | |  |  |  |  |  |  |  |
| oxidation-reduction process (10-5, 26 genes) | | | | |  |  |  |  |  |  |  |  |
| organonitrogen biosynthetic process (0.0002, 23 genes) | | | | | | |  |  |  |  |  |  |
| cell wall organization or biogenesis (0.0051) | | | | |  |  |  |  |  |  |  |  |
| *ECM33* | *PST1* | *CWP2* | *SSP1* | *BGL2* | *BIT61* | *OSW2* | *BCH1* | *KTR2* | *PIR3* | *YPS3* | *SPS100* | *YPS1* |
| *SPO73* | |  |  |  |  |  |  |  |  |  |  |  |
| sexual reproduction (0.0702) | | | |  |  |  |  |  |  |  |  |  |
| *MFA2* | *TUB2* | *GPA2* | *SSP1* | *SCP160* | *MF(α)1* | *EMI2* | *SPO16* | *OSW2* | *PRM7* | *SPS100* | *SPO73* | *CLN2* |
| regulation of transcription (1) | | | |  |  |  |  |  |  |  |  |  |
| *SWI5* | *CSE2* | *PCL9* | *CBF1* | *NPL3* | *VPS36* | *ABF1* | *EMI2* |  |  |  |  |  |
| Derepressed in Q cells (82 known genes) | | | | |  |  |  |  |  |  |  |  |
| cellular process (0.0397, 74 genes) | | | |  |  |  |  |  |  |  |  |  |
| proteolysis (1) | |  |  |  |  |  |  |  |  |  |  |  |
| *YGK3* | *JEM1* | *ADD37* | *PRE6* | *PCP1* | *CDC27* | *YPS3* | *APC1* | *RPN10* | |  |  |  |
| cell cycle (1) | |  |  |  |  |  |  |  |  |  |  |  |
| *BFA1* | *BUD3* | *DPB2* | *BIK1* | *CNN1* | *SIT4* | *IPL1* | *MEI4* | *CDC39* | *MPC54* | *CDC27* | *CEF1* | *SML1* |
| *APC1* |  |  |  |  |  |  |  |  |  |  |  |  |
| regulation of transcription (1) | | | |  |  |  |  |  |  |  |  |  |
| *DPB2* | *YGK3* | *STD1* | *ECM22* | *BDP1* | *GSM1* | *GAL4* | *CDC39* | *YRM1* | *BDF2* | *DAL80* |  |  |
| Organized by GO process. Corrected P value for enrichment and gene number is shown in parentheses. | | | | | | | | | | | |  |
