# Supplementary material for: Xbp1 Directs Global Repression of Budding Yeast Transcription during the Transition to Quiescence and Is Important for the Longevity and Reversibility of the Quiescent State
Source: PLoS Genet. 2013 Oct 31;9(10):e1003854. doi: 10.1371/journal.pgen.1003854 (PMC3814307; doi:10.1371/journal.pgen.1003854)
Supplement: Table S4 — PCR primers used in chromatin immunoprecipitations. (DOCX) [file pgen.1003854.s005.docx]

|  | Supplemental Table S4: Oligonucleotides used in ChIP |  |
| --- | --- | --- |
|  | Oligonucleotides | Gene Promoter |
| BL336 | cgctgtgctcaaaccctaaattgt | *CLN3* -653 F |
| BL337 | tttgactggcagactcagtagtag | *CLN3* -314R |
| BL670 | CTGTCAGAATATGGGGCCGTAG | *ARS504* +933F |
| BL671 | CACCCCGAAGCTGCTTTCAC | *ARS504* +1078R |
| BL2236 | GTCAGGATGTAAGAACTACG | *DOG2* -282F |
| BL2237 | TGTATCGATAACGTGTTGGC | *DOG2* -50R |
| BL2238 | GAAGTACCGATGAGATGAGA | *PIS1* -270F |
| BL2239 | ACTTGAAGGCTCCACAGTGA | *PIS1* -54R |
| BL2240 | CACATTGGCCGTTTTAACGC | *AFT1* -520F |
| BL2241 | CTATCCGAGGTTGTGTACTG | *AFT1* -280R |
| BL2242 | GCAGTGGTAGGCACAATTTTG | *CDC10* -330F |
| BL2243 | TTCTTGTCTTTGCCTGAAAAAAG | *CDC10* -50R |
| BL2244 | CCCTCAAGTTTAGAAAATGCC | *NRG2* -920F |
| BL2245 | CTCCTTTTTTTCTTGGCTTCG | *NRG2* -721R |
| BL2246 | CCTCCTCTCTCCATTGTTCT | *RFX1* -750F |
| BL2247 | GAGGTGTAGGGTAAATCCCA | *RFX1* -530R |

F denotes forward, R denotes reverse primers
